# Regression/Eradication of gliomas in mice by a systemically-deliverable ATF5 dominant-negative peptide

**DOI:** 10.18632/oncotarget.7212

**Published:** 2016-02-05

**Authors:** Charles C. Cates, Angelo D. Arias, Lynn S. Nakayama Wong, Michael W. Lamé, Maxim Sidorov, Geraldine Cayanan, Douglas J. Rowland, Jennifer Fung, Georg Karpel-Massler, Markus D. Siegelin, Lloyd A. Greene, James M. Angelastro

**Affiliations:** ^1^ Department of Molecular Biosciences, University of California, Davis School of Veterinary Medicine, Davis, CA, USA; ^2^ Center for Molecular Genomic Imaging, Davis, CA, USA; ^3^ Department of Pathology and Cell Biology, Columbia University, New York, NY, USA; ^4^ Cedars-Sinai Medical Center, Los Angeles, CA, USA; ^5^ Moores-UCSD Cancer Center, La Jolla, CA, USA

**Keywords:** cell penetrating peptide, ATF5, d/n- ATF5, apoptosis, brain cancer

## Abstract

Malignant gliomas have poor prognosis and urgently require new therapies. Activating Transcription Factor 5 (ATF5) is highly expressed in gliomas, and interference with its expression/function precipitates targeted glioma cell apoptosis *in vitro* and *in vivo*. We designed a novel deliverable truncated-dominant-negative (d/n) form of ATF5 fused to a cell-penetrating domain (Pen-d/n-ATF5-RP) that can be intraperitoneally/subcutaneously administered to mice harboring malignant gliomas generated; (1) by PDGF-B/sh-p53 retroviral transformation of endogenous neural progenitor cells; and (2) by human U87-MG xenografts. *In vitro* Pen-d/n-ATF5-RP entered into glioma cells and triggered massive apoptosis. *In vivo*, subcutaneously-administered Pen-d/n-ATF5-RP passed the blood brain barrier, entered normal brain and tumor cells, and then caused rapid selective tumor cell death. MRI verified elimination of retrovirus-induced gliomas within 8-21 days. Histopathology revealed growth-suppression of intracerebral human U87-MG cells xenografts. For endogenous PDGF-B gliomas, there was no recurrence or mortality at 6-12 months *versus* 66% mortality in controls at 6 months. Necropsy and liver-kidney blood enzyme analysis revealed no adverse effects on brain or other tissues. Our findings thus identify Pen-d/n-ATF5-RP as a potential therapy for malignant gliomas.

## INTRODUCTION

Gliomas are the most common primary malignant brain tumors and many are, or develop to become, particularly invasive. Despite advances, there has been limited progress in improving patient outcomes and for this reason additional effective treatment approaches are urgently needed [1, 2]. Activating transcription factor 5 (ATF5; activating transcription factor/CREB family member) is a potential target for treatment of gliomas [3–6]. ATF5 is highly expressed in human glioblastomas [3] with expression levels reported to inversely correlate with disease prognosis [6, 7]. Gliomas are thought to arise from several cell origins. It is additionally relevant that ATF5 is also expressed by neural and glial progenitor cells [8–10], CD133+ glioma stem cells [11], and by stem cells isolated from human glioblastomas [6].

Interference with ATF5 function by a dominant negative-form of the protein promotes massive apoptosis of glioblastoma cells *in vitro* and *in vivo* without detrimental effect on normal cells [3, 4, 8–10, 12]. ATF5 silencing with siRNA *in vitro* achieved similar results, indicating that these effects were due to ATF5 loss-of-function [3]. Recently, we demonstrated the efficacy of d/n-ATF5 in a bi-transgenic TET-off mouse model in which this construct was conditionally induced under regulation of the glial-fibrillary protein promoter. Expression of d/n-ATF5 caused complete regression/eradication of gliomas induced from endogenous progenitor cells and did so without damage to normal brain tissue [4]. The basis for the role of ATF5 in survival of tumor cells is not completely understood, but mechanistic studies have suggested that ATF5 supports glioblastoma cell survival by regulating expression of the anti-apoptotic proteins MCL1 [6] and Bcl2 [13] and of the Egr-1 gene [14].

In the present study, we sought to design and test a form of d/n-ATF5 that can be effectively delivered to glioma cells in the brain. We achieved this by generating a truncated, but fully active form of the peptide fused to a cell-penetrating domain that permits passage through the blood-brain barrier and into intact cells. We report that this agent, when delivered systemically to adult mice with gliomas generated from endogenous neuro-progenitor cells or intracerebral human U87-MG xenografts, causes long-term regression/suppression of the tumors as shown by MRI and histopathology.

## RESULTS

### Generation of a cell-penetrating form of d/n-ATF5

**Figure 1:**
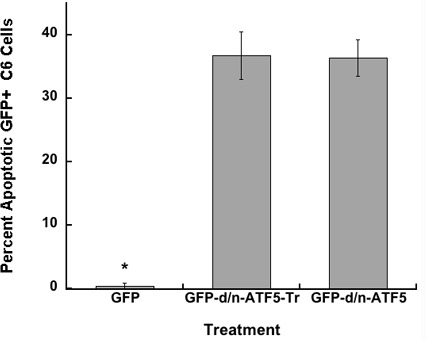
GFP-d/n-ATF5 C-terminally truncated fusion protein (GFP-d/n-ATF5-Tr) promotes the same level of apoptosis as full-length GFP-d/n-ATF5 protein in C6 glioma cells C6 cells were transfected with *pQC-X-I-eGFP*, *pQC-d/n-GFPATF5*, or *pQC-GFPATF5-tr*. The percentages (mean ± SEM, *n* = 4; total of approximately 200 cells scored per condition) of condensed apoptotic nuclei in GFP + transfected cells were determined 2 days later. Student's *t*-test; GFP+ cells *versus* GFP-d/n-ATF5+ cells or GFP-d/n-ATF5-tr cells, (*p < 0.05); GFP-d/n-ATF5+ cells *versus* GFP-d/n-ATF5-tr cells, (Not Significant).

Given the successful regression/eradication of endogenously-formed gliomas achieved by regulated expression of d/n-ATF5 in mouse brain, we devised a modified cell-penetrating form of this peptide for systemic delivery capable of reaching widely dispersed tumor cells through the advantage of rapid biodistribution, reduced immune response, with the ability to pass through the blood brain barrier into neural cells [4]. Our original d/n-ATF5 is an N- terminally truncated form of ATF5 that includes the wild-type leucine zipper domain with an amphipathic α-helical sequence with leucine repeats at every seventh residue replacing the DNA binding domain [8]. The enhanced leucine zipper region permits interaction with ATF5, but not with DNA, and consequently acts as an effective d/n suppressor of ATF5 actions [8, 15]. N-terminal domain deletion substantially stabilizes d/n-ATF5 against degradation [12, 16]. To design a deliverable form of d/n-ATF5, we first truncated the last 25 amino acids of the protein, which included the C-terminal two valine/valine heptad repeats. Structural studies suggest that truncation of this region may reduce aggregation at body temperatures [17]. Transfection of this deleted construct into C6 glioblastoma cells showed equal effectiveness as the full length d/n-ATF5 in promoting apoptosis (Figure 1; *p* < 0.05).

**Figure 2:**
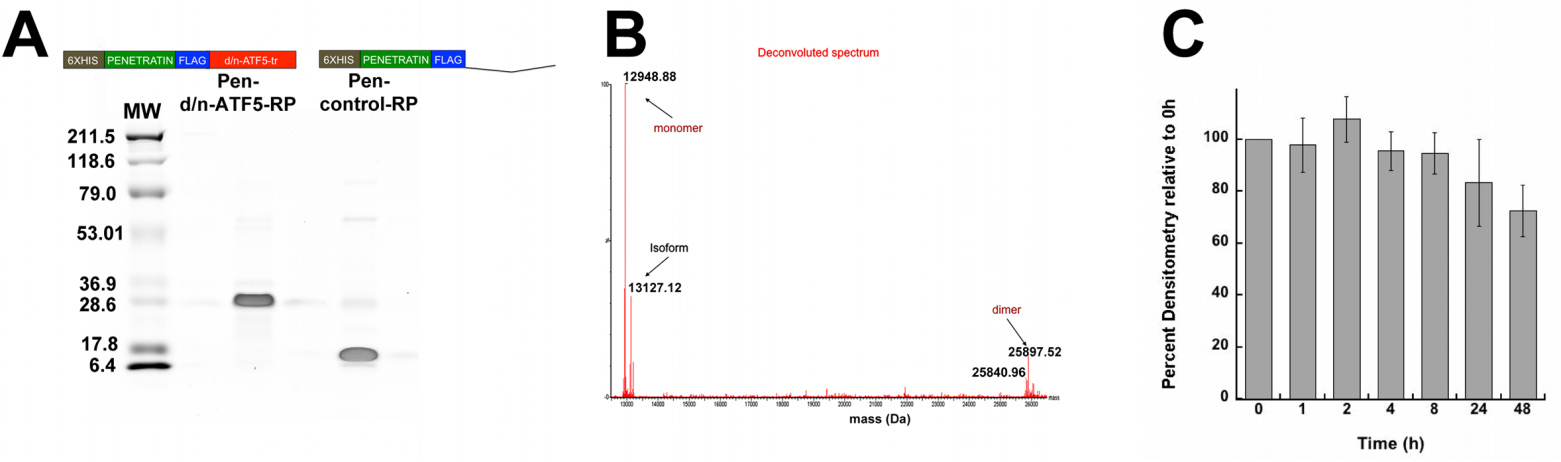
Purity and molecular properties of bacterially expressed and purified 6xhistidine-Flag-Tagged Penetratin-Flag-D/N-ATF5-tr (Pen-d/n-ATF5-RP) and 6xhistidine-Flag-Tagged Penetratin-Flag-Control (Pen-control-RP) peptides **A.** Coomassie stained SDS-PAGE of purified Pen-d/n-ATF5-RP and Pen-control-RP (5 μg per lane). Molecular weight markers are shown on the left, and a linear scheme of each peptide is shown above each lane. Purification was as described in Methods. **B.** Deconvoluted mass spectra from LC-high Resolution mass spectrometry of purified Pen-d/n-ATF5-RP. The most abundant species is the 12,948.88 Da monomer form without formyl-methionine followed by the formyl-methionine 13,127 Da monomer form (isoform). The spectrum also reveals a small amount of the 25,897.5 Da dimer. **C.** Stability of Pen-d/n-ATF5-RP in Human Serum. Pen-d/n-ATF5-RP (36 μM) was incubated with human serum (25% v/v in PBS) at 37° C for 0 to 48 h. Aliquots were withdrawn at various times and the Pen-d/n-ATF5-RP peptide was resolved by SDS-PAGE, transferred to PVDF membrane and probed with anti-Flag antibody. The anti-Flag signal was detected by near IR using LiCor software and densitometry of the band at the expected size of Pen-d/n-ATF5-RP and quantified using Image J. Values are mean ± SEM, *n* = 3).

We designed the cell-penetrating form of the C-terminally truncated Flag-tagged- d/n-ATF5 (d/n-ATF5-tr) by N-terminally fusing Flag-tagged d/n-ATF5-tr to a 6x histidine repeat, followed by a penetratin sequence (Figure 2A). Penetratin sequence is a 16-amino acid motif from the Antennapedia homeodomain protein permitting passage of fused cargos through biological membranes into cells [18]. Milligram quantities of the protein (designated Pen-d/n-ATF5-Recombinant Protein (RP)) were generated by expression in bacteria followed by purification by cobalt resin affinity chromatography using the 6xHis sequence. SDS-PAGE showed the purified preparations were more than 95% homogeneous with minor species including what appeared to be aggregated protein multimers. Calculated Mr of Pen-d/n-ATF5-RP with normal bacterial removal of the N-formylmethionine is 12,949.18 Da, but the major purified product shows an apparent molecular mass between 25-28 KDa by SDS-PAGE (Figure 2A). Wild type ATF5 and the ATF5 leucine zipper can migrate anomalously when subjected to SDS-PAGE [19, 20], and high resolution LC-HRMS verified the correct molecular weight of Pen-d/n-ATF5-RP while in its solution state. The deconvoluted spectra revealed the most abundant form to be the predicted 12,948.7 Da monomer, with a low amount of dimer at 25,897.5 Da (Figure 2B). Prior studies have also shown that recombinant wild type full-length ATF5 or the bzip domain of ATF5 can form dimers *in vitro* [19–21]. Because multi-isomers produced a range of MW of the monomer, we elected to use the computed MW of 13080,which includes the methionine without formyl group for our studies. Finally, as a control for Pen-d/n-ATF5-RP, we generated by similar means a peptide (Pen-Control-RP) that lacks the d/n-ATF5-tr sequence (Figure 2A). The purified recombinant control (with a calculated molecular mass of 7,099.98 Da) migrated at an apparent MW of 7,100 Da by SDS-PAGE (Figure 2A).

Because Pen-d/n-ATF5-RP is designed for systemic administration, we showed stability in presence of human serum at 37° C with no significant degradation at 8 h and a mean loss of 28% of full-length protein by 48 h (Figure 2C).

### Pen-d/n-ATF5-RP rapidly enters and causes apoptosis of cultured glioblastoma cells

**Figure 3:**
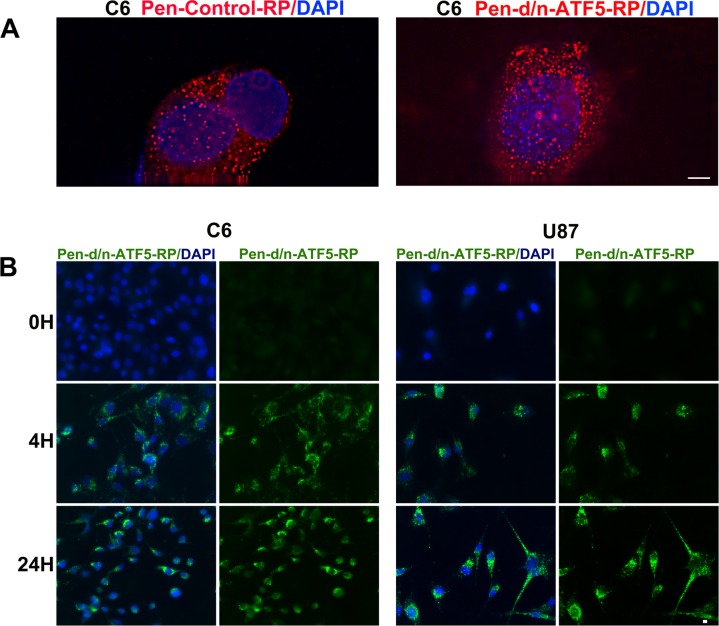
Uptake and retention of Pen-d/n-ATF5-RP by cultured glioblastoma cells **A.** Confocal images of C6 rat glioblastoma cells incubated for 4 hours with either 200 nM Pen-control-RP (left) or Pen-d/n-ATF5-RP (right). Cells were washed, fixed and stained with anti-Flag (red) and DAPI (blue). Scale bar = 2 μm. **B.** Rat C6 and human U87 glioblastoma cells were incubated for the indicated times with 3 μM Pen-d/n-ATF5-RP, washed, fixed and immunostained with anti-Flag (green) and DAPI (blue). Scale bar = 5 μm.

Before carrying out animal experiments, we verified that Pen-d/n-ATF5-RP enters and kills glioblastoma cells in culture. When added to serum-containing cultures of rat C6 and human U87 glioblastoma cells, both Pen-control-RP and Pen-d/n-ATF5-RP were readily detectable in the cells within 2-4 h and remained detectable for at least 24 h (Figure 3A, 3B). Confocal microscopy revealed that the peptides were present in both the cytoplasmic and nuclear compartments (Figure 3A).

**Figure 4:**
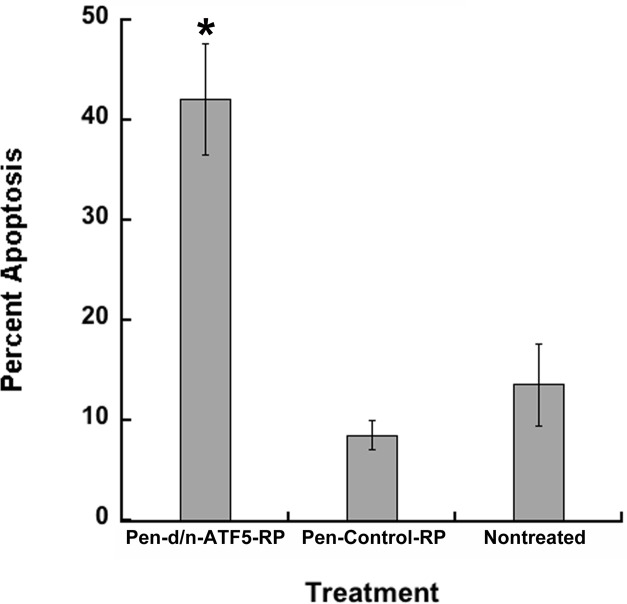
Pen-d/n-ATF5-RP promotes apoptosis of C6 glioblastoma cells C6 cells were treated with 3 μM Pen-d/n-ATF5-RP or 3 μM Pen-Control-RP, or were untreated. The percentage (mean ± SEM; *n* = 4 in 2 independent experiments; approximately 200 cells scored) of condensed apoptotic nuclei in cells was determined 5 days later. Student's *t*-test; Pen-d/n-ATF5-RP *versus* Pen-Control-RP cells or nontreated, (*p < 0.05); Pen-Control-RP cells *versus* nontreated cells, (*p* = 0.29).

C6 cultures exposed to Pen-control-RP and Pen-d/n-ATF5-RP were also assessed for apoptotic cell death. Pen-Control-RP treated cultures showed background levels of apoptotic death similar to that in non-treated cultures, whereas cultures treated with Pen-d/n-ATF5-RP showed greatly increased numbers of dying cells (Figure 4; *p* < 0.05). These actions are similar to what others and we have previously reported for multiple rodent and human glioblastoma cell lines transfected with d/n-ATF5 constructs or exposed to ATF5 siRNA [3, 4, 6, 13].

### Systemically-delivered Pen-d/n-ATF5-RP crosses the blood brain barrier, enters cells and selectively triggers rapid, selective apoptotic death of PDGF-BHA/shp53 induced glioma cells

To test the capacity of Pen-d/n-ATF5-RP to reach and treat primary brain tumors, we used a model in which gliomas are generated by stereotactic injection of PDGF-B-HA/shRNA-p53 retrovirus into the adult mouse brain. The tumors are presumably derived from endogenous dividing progenitor cells and closely resemble infiltrative human gliomas ranging from grades II-IV [4, 22–28]. The tumors were detectable as early as 52 days post-injection by MRI (see below) and were histologically identifiable by the presence of the HA tag as well as by high cellularity, hyperchromatic nuclei, and elevated Ki67 staining.

**Figure 5:**
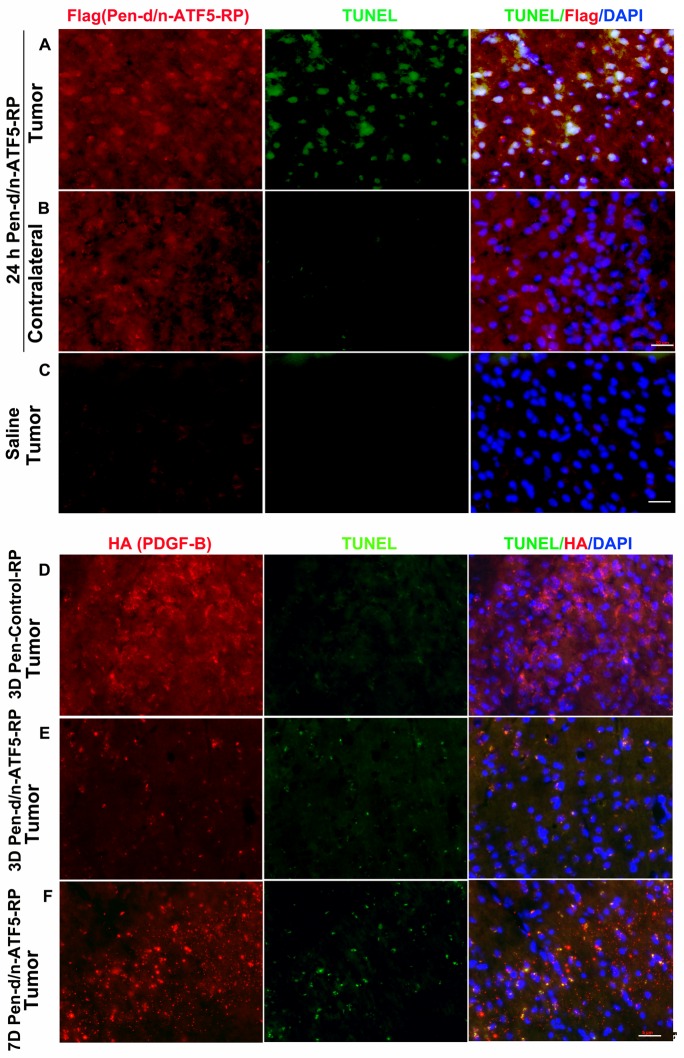
Pen-d/n-ATF5-RP enters the mouse brain and causes targeted apoptosis of glioma cells **A.**-**F.** Representative brain sections stained with Flag antibody to indicate presence of Pen-d/n-ATF5-RP or HA to identify presence of tumor-inducing retrovirus (red); TUNEL to identify apoptosis (green) and DAPI to localize nuclei (blue). **A.** Murine brain tumor 24 h post-treatment (16 h after last injection) with Pen-d/n-ATF5-RP (52 days post-retrovirus injection). **B.** Normal contralateral cerebral hemisphere of the same mouse in **A.**. **C.** Murine brain tumor 24 h post-injection with saline (59 days post-retrovirus injection). Presence of Pen-d/n-ATF5-RP within cells is confirmed in the treated mouse **A.**,**B.** *versus* saline control **C.** by increased Flag antibody staining. Glioma cell-specific induction of apoptosis by Pen-d/n-ATF5-RP is illustrated by increased TUNEL staining (green) in **A.** as compared to **B.** and **C.**. **D.** TUNEL and DAPI staining of a tumor-containing brain section 160 days post-retrovirus injection and 3 days after injection of Pen-control-RP. Note HA+ cells identifying tumor cells and absence of TUNEL staining. **E.** Staining as in **D.** of a tumor-containing section (143 days post-retrovirus injection) and 3 days [3D] after Pen-d/n-ATF5-RP treatment. Note the presence of TUNEL staining in HA+ tumor cells and fragmented appearance of the staining as compared to **A.** and **D.**. **F.** Staining as in **D.** of a tumor-containing section 150 days after retrovirus injection and 2 days after 2 treatments of subcutaneous Pen-d/n-ATF5-RP injections at seven days [7D] after the first injection. Note the qualitative similarity of staining pattern to **E.** with fragmented PDGF-B-HA and TUNEL staining. Scale bars equal 20 μm.

In an initial set of experiments, Pen-d/n-ATF5-RP, saline or Pen-Control-RP was delivered intraperitoneally to tumor-bearing mice in a single set of four injections each of 1 mg/kg at intervals of 1-2 h. The mice were sacrificed 16-64 hours after the last injection and the fixed brains were stained with anti-Flag antibody to detect Pen-d/n-ATF5-RP or with anti-HA to mark PDGF-B-HA expressing tumor cells, and for TUNEL to identify dying cells. At 16 h, both tumor and normal brain cells (in the contralateral hemisphere from the tumor) showed Flag staining indicative of extensive uptake of Pen-d/n-ATF5-RP; there was no signal with saline injection (Figure 5A-5C). Flag staining was still evident at 40 h after treatment and was detectable, though at reduced levels at 64 h (Supplementary Figure 1; *p* < 0.05). While normal brain tissue showed no TUNEL staining (Figure 5B), there was extensive TUNEL staining within the tumors one day after treatment with Pen-d/n-ATF5-RP (Figure 5A). Little or no TUNEL signal was observed in tumors of animals treated with saline (Figure 5C). Co-localized TUNEL and PDGF-B-HA+ tumor marker staining continued to be evident at 64 h after Pen-d/n-ATF5-RP treatment, but the signals indicated cell degeneration and fragmentation (Figure 5E) compared with cells treated with this peptide for 16 h (Figure 5A) or with Pen-Control-RP peptide (Figure 5D).

To enhance the potential long-term therapeutic efficacy of Pen-d/n-ATF5-RP administration in the PDGF-BHA/p53 mouse tumor model, we devised a treatment protocol in which tumor-bearing animals received two sets of subcutaneous injections, 5 days apart, each as described above. Tumors of mice assessed two days after the second treatment (7 days after initial treatment) showed patterns of HA and TUNEL staining, that, similar to 64 h after a single set of treatments, indicated cell degeneration and fragmentation (Figure 5F).

Full body necropsy of non-tumor bearing animals one (*n* = 2) or two days (*n* = 2) after completion of the above dual treatment regimen revealed no evident pathological lesions to internal organs and no evident abnormalities of the cerebrum or cerebellum (Supplementary Figure 2 and Supplementary Table 1). In addition, a liver-kidney serum chemistry panel carried out 1 day after the second set of Pen-d/n-ATF5-RP injections indicated no damage to either organ (Supplementary Table 1; *n* = 2)

### Systemically delivered Pen-d/n-ATF5-RP promotes rapid regression of mouse PDGF-B/p53 induced gliomas without recurrence and suspension of U87-MG/Luciferase xenografts growth

**Figure 6:**
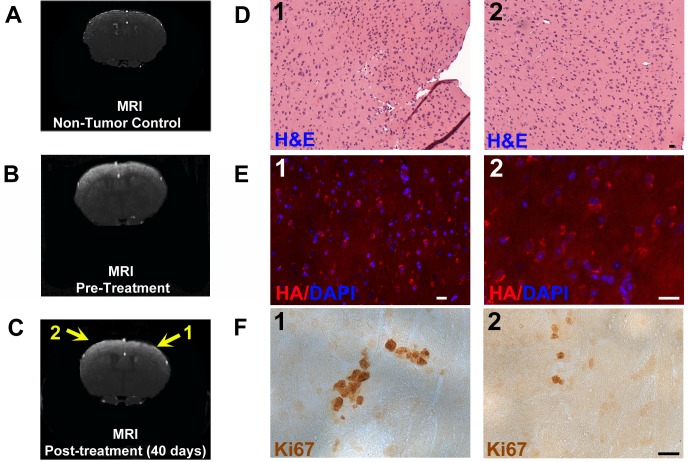
Example of MRI and histopathology of a mouse glioma treated with Pen-Control-RP peptide **A.** Post-contrast 3D FLASH MRI coronal image of the cerebrum of a control mouse that was not injected with PDGF-B-HA/sh-p53 retrovirus. **B.** Post-contrast 3D FLASH MRI coronal image of mouse cerebrum showing a bilateral tumor (white contrast) 246 days after PDGF-B-HA/shp53 retrovirus injection and prior to treatment with Pen-Control-RP peptide. **C.** Post-contrast 3D FLASH MRI image of the same mouse brain 40 days after subcutaneous treatment with Pen-Control-RP peptide (as described in the text) reveals persistence of the tumor (arrows). **D.** H&E stained sections of the same mouse brain at tumor-containing areas 1 and 2 shown by arrows in panel **C.**. The mouse was sacrificed 116 days after the second treatment with Pen-Control-RP peptide due to moribund behavior. Presence of tumor is indicated in both sections by hyperchromatic nuclei and higher cellularity. **E.** Immunostaining for HA tag in sections from areas 1 and 2 shown in Panel **C.** reveals presence of virally-delivered PDGF-B-HA in induced tumor cells. **F.** Immunostaining of sections from areas 1 and 2 shown in Panel **C.** reveals a high index of Ki67+/dividing cells indicative of tumor. Scale bars in D-F are 20 μm.

**Figure 7:**
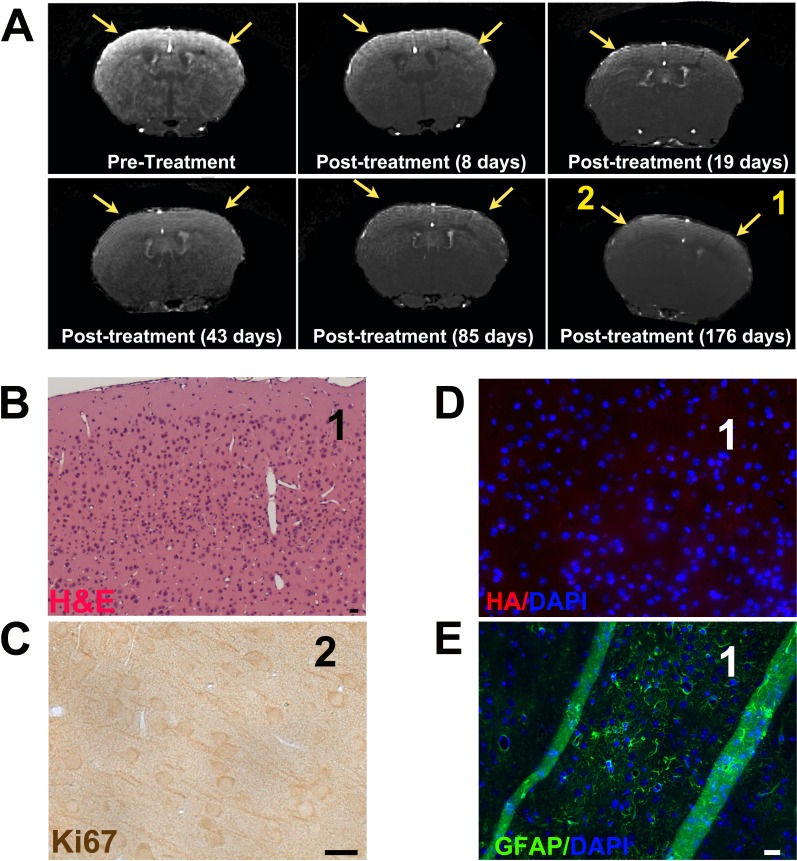
Pen-d/n-ATF5-RP promotes rapid and long-term regression/eradication of mouse glioma as indicated by MRI and histology **A.** Post-contrast 3D FLASH MRI scans of a mouse brain before and at various times after treatment (as described in text) with Pen-d/n-ATF5-RP. Pretreatment shows image of cortex 243 days after PDGF-B-HA/shp53 retrovirus injection. Yellow arrows indicate location of the bilateral tumor. Post-treatment images of the same position of the mouse cortex are at the indicated times after the second administration of Pen-d/n-ATF5-RP. Yellow arrows in post-treatment images show location of original tumor. **B.** H&E image of the same mouse brain harvested 192 days after the second Pen-d/n-ATF5-RP treatment. Region 1 represents the location of the section as shown in the final time point in **A.** and at which the tumor was present before treatment. Note the absence of hyperchromatic nuclei and higher cellularity that characterize gliomas. **C.** Ki67 staining in region 2 (from Panel A/176 days post-treatment). Note the absence of Ki67+/proliferating cells seen in gliomas. **D.** HA/DAPI staining of section from region 1. Note the absence of cells expressing exogenous PDGF-B-HA. **E.** GFAP/DAPI staining of section region 1. Note clusters of GFAP+ cells consistent with the presence of a glial scar where the tumor was formerly present. Lack of HA staining of a nearby section confirmed the absence of tumor cells. Diagonal green stripes are due to tissue folds. Scale bar is 20 μm.

We next assessed whether systemic administration of Pen-d/n-ATF5-RP promoted prolonged regression of gliomas in our mouse model. To achieve this we used MRI (post-contrast enhanced 3D FLASH T1 weighted) to assess tumors before and at various times after treatment with Pen-d/n-ATF5-RP, Pen-control-RP or no treatment. In many cases, the tumors were either multifocal or present in both hemispheres prior to treatment (Figures 6, 7, Supplementary Figures 3, 4). The peptides were injected subcutaneously using the two treatment protocol described above. Treatments commenced only after the presence of tumors was verified by MRI and were randomly assigned.

As anticipated, in no case did we observe tumor regression as assessed by MRI in untreated animals (*n* = 5) or animals treated with Pen-control-RP (*n* = 4). A typical example for an animal treated with control peptide is shown in Figure 6. Tumor presence was verified by histology on brains of animals that either died or were sacrificed after exhibiting moribund behavior or that survived beyond the study endpoint (6 months after MRI tumor detection). The tumors were HA+ (Figures 6E, Supplementary Figure 3), indicating the presence of the tagged PDGF-B and exhibited hyperchromatic nuclei (Figure 6D) and elevated Ki67 staining typical of gliomas (Figure 6F). The infiltrative tumor boundaries matched those in the MRI images (Figure 6).

**Figure 8:**
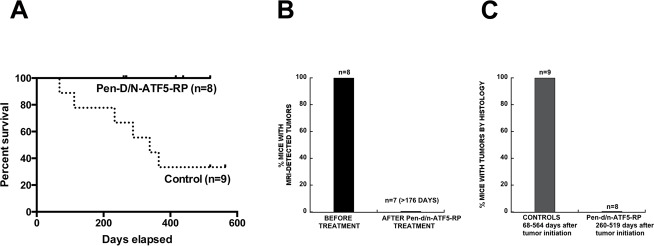
Long-term survival and tumor presence outcomes for PDGF-BHA/shp53 glioma-bearing mice treated with Pen-d/n-ATF5-RP **A.** Survival of glioma (PDGF-B/shRNA-p53 induced)-bearing mice (verified by MRI) with or without treatment with Pen-d/n-ATF5-RP (subcutaneous delivery as described in the text). Of the nine control mice, four mice were treated with Pen-Control-RP peptide and five were untreated. The experimental endpoint was 200 days after initial tumor detection by MRI. Survival analysis achieved by Log-rank (Mantel-Cox) test showed a *p*-value = 0.0194. **B.** MRI outcomes for tumor-bearing mice before and after subcutaneous treatment with Pen-d/n-ATF5-RP as described in the text. Fisher Exact Match test showed *p* = 0.0002 with Positive Predictive Value 95% confidence interval of 0.6306 to 1.000. The latter times range from 176-225 days after tumor treatment (183-230 days after tumor detection). **C.** Brain histopathological outcomes for tumors in control and Pen-d/n-ATF5-RP treated mice. In all cases, MRI verified the presence of tumors prior to treatment. Fisher Exact Match test showed *p* < 0.0001 with Positive Predictive Value 95% confidence interval of 0.0 to 0.3694. The brains of animals described in **A.** were harvested either after death (6 controls), after the experimental endpoint (4 Pen-d/n-ATF5-RP treated animals; 2 Pen-Control or 1 non-Treated animal) or after sacrifice for non-tumor related health problems (2 Pen-d/n-ATF5-RP treated animals). For treated animals, histological analysis was carried out 260-547 days after tumor initiation (183-392 days after Pen-d/n-ATF5-RP administration and 190-397 days after initial tumor detection). Brain sections were prepared as described in Methods and were stained with H&E and immunostained for Ki67 and HA (to identify PDGF-B-HA+ tumor cells). The presence/absence of tumors was based on observations of hyperchromatic nuclei, high cellularity, elevated Ki67 staining and HA immunostaining.

For mice treated with Pen-d/n-ATF5-RP, MRI revealed significant reduction (2/5; Figure 7, Supplementary Figure 4) or un-detectability (3/5) of tumor signals at 8 days after treatment (the earliest time monitored) and full loss of detectable tumor signal within 3 weeks (*n* = 7/7). When assessed by MRI at 176-225 days after peptide treatment, 7/7 mice assessed were tumor-free (see for example, Figure 7; Supplementary Figure 4; and Figure 8B with *p* = 0.0002). Thus, Pen-d/n-ATF5-RP treatment appeared to rapidly clear gliomas without MRI-detectable recurrence for at least 6-13 months.

Postmortem histology (*n* = 8; 183-392 days after treatment; 190-397 days after tumor detection) corroborated the MRI findings of tumor regression/eradication (Figures 7, 8C with a *p* < 0.0001, Supplementary Figure 4). As in the rest of the brain, areas that initially had been tumor positive by MRI, showed an absence of hyperchromatic nuclei or high cellularity or elevated Ki67 staining (Figure 7; Supplementary Figure 4). There was also no staining (other than scarce scattered single cells) for PDGF-B-HA+ (Figure 7; Supplementary Figure 4). There were however, foci of GFAP+ cells, suggesting glial activation and scarring in the areas where tumors had been present (Figure 7; Supplementary Figure 4).

Human U87-MG-Luc2 xenografts formed intracranial tumors that were both intra-cerebral and exophytic (extra-cerebral). Subcutaneous (4 mg/kg) Pen-d/n-ATF5-RP, with scheduling varying from two doses at 5 days apart to weekly resulted in either the absence or significant loss of volume of intracerebral tumors compared to Pen-Control-RP (Supplementary Figure 5). The exophytic tumors appeared not to be affected by either treatment regimen resulting in the absence of increased survival time. Cerebrum, with greater enriched vascularization, provides an environment that both accelerates tumor grow but also results in a higher therapeutic bio-distribution for the peptide. Extra-cranial environments have potentially poorer tumor vascularization resulting in decreased uptake of therapeutics. Brain tumor xenografts can have as much as 50% more vascular network in the brain than subcutaneous xenografts for the identical cell line [29–31].

### Systemically delivered Pen-d/n-ATF5-RP promotes survival while maintaining normal brain and tissue integrity

All eight retrovirus PDGF-B induced tumor-bearing mice treated with Pen-d/n-ATF5-RP survived to the nominal 180 day endpoint of the study after detection of tumors (Figure 8A; Log-rank (Mantel-Cox) test *p* = 0.0194). In contrast, 6/9 control mice died within this time. In our past study 40% (*n* = 16) of mice control” died within 180 days of tumor initiation [4].

Other than the absence of tumors and the presence of glial scarring in areas of prior tumor localization, H&E staining of the brains of the C57BL/6 mice sacrificed 6-13 months after Pen-d/n-ATF5-RP treatment indicated no evident abnormalities and both the subventricular and hippocampal subgranular zones appeared normal (Supplementary Figure 2). Additionally, the weights of the treated C57BL/6 mice prior to sacrifice were either within (4/7) or greater than (3/7) one standard deviation of the mean weight of age-matched controls given in the Mouse Phenome Database at the Jackson Laboratory (http://phenome.jax.org/db/q?rtn = strains/details&strainid = 7). Two C57BL/6 mice were also subjected to full body necropsy at > 6 months of treatment (190 days and 183 days, corresponding to mice with eradicated tumors in Figures 7, and Supplementary Figure 4, respectively). No pathological changes were seen in any of the organs surveyed (Supplementary Table 1).

Finally, Pen-d/n-ATF5 is able to promote apoptosis in glioma stem cell lines or so-called “tumor-initiating cells” [6]. In each case, the population of stem cells entered into early (Annexin V +) and late phase apoptosis (Annexin V+ and propidium iodide+) with Pen-d/n-ATF5-RP or chemical synthesized Pen-d/n-ATF5-SYN (lacking Flag-tag) compared to recombinant Pen-Control-RP (Supplementary Figure 6).

## DISCUSSION

A variety of observations have identified ATF5 as a potential target for treatment of malignant gliomas. [3–7, 32] ATF5 is highly expressed by glioblastoma as well as by glioblastoma stem cells; ATF5 expression in glioblastoma inversely correlates with prognosis; and interference with ATF5 expression or activity causes death of malignant glioma cells *in vitro* and *in vivo*. To interfere with ATF5 function, we previously designed and employed a d/n-ATF5 construct delivered by viral infection, transfection or as an inducible transgene. Although this supported the potential of ATF5 as a therapeutic target, such a reagent was not a practical means to affect ATF5 function in a clinically relevant setting. To convert d/n-ATF5 into a deliverable therapeutic, we generated a truncated form fused to a penetratin cell-penetrating domain. A variety of previous studies have supported the potential utility of cell-penetrating peptides for therapeutic purposes [18, 33–35]. Our findings show that Pen-d/n-ATF5-RP enters and promotes apoptotic activity in cultured glioblastoma cells, including glioma stem cells, and that when systemically administered to animals, crosses the blood brain barrier, enters brain and tumor cells and causes massive tumor cell death and long-term tumor regression/eradication without apparent harm to normal tissues. Moreover, the demonstration that Pen-d/n-ATF5 as a recombinant or a chemical synthetic peptide triggers death in glioma stem cells substantiates the potential of its therapeutic potential to eradicate both the non-stem cell glioma and the glioma stem population to reduce the opportunity of recurrence.

Another key feature of our study was that the treated tumor-bearing animals survived for at least 6-13 months. By contrast, 2/3 of control animals died or showed morbidity within 189 days of tumor detection and all were tumor positive at death or at the 6-month point. Pen-d/n-ATF5-RP regimen for mice with U87-MG-Luc2 intracranial xenografts revealed smaller volumes or the absence of intracerebral tumors. This data are relevant to treatment of clinical human spontaneous tumors that do not metastasize outside the CNS.

The model in which malignant gliomas were induced in adult mice by retrovirally expressed PDGF-B and p53 shRNA is derived presumably by transformation of PDGF-α-receptor+ neural progenitors and oligodendrocyte precursors. Such tumors resemble high grade human glioma [4, 22–28] and, like the latter, are highly diffuse, relatively large and can invade both hemispheres. Given the wide expression of ATF5 in human glioblastomas and lower grade gliomas and the variety of human and rodent-derived glioblastoma cell lines (with and without compromised p53 and PTEN) that express and require ATF5 for survival [3, 4, 6, 7], it seems likely that a range of malignant glioma cell types will be susceptible to cell-penetrating d/n-ATF5.

An important aspect of our study was that although Pen-d/n-ATF5-RP promoted regression/eradication/suppression of tumors, it had no apparent adverse effects on normal tissue. While one cannot currently completely rule out side effects of the treatment, it is significant that treated animals survived without apparent effect for at least 6-13 months and that no evident acute or long term tissue damage was observed. In addition, any potential negative effects of Pen-d/n-ATF5-RP may be mitigated by the limited duration of treatment. Because the present supplies of bacterially-produced Pen-d/n-ATF5-RP are limited, we have not carried out systematic dose-response and dosing studies in either glioma animal model.

Although we focus here on malignant gliomas, it is significant to note that ATF5 is expressed by a wide variety of carcinomas [32, 36–39], and that culture studies have shown apoptotic actions of d/n-ATF5 or ATF5 siRNA on tumor cells from a diverse range of tissues. [32, 36, 39, 40] This raises the possibility that the therapeutic potential of cell penetrating forms of ATF5 may not be limited to gliomas.

## MATERIALS AND METHODS

### Retrovirus-induced mouse glioblastoma and U87-MG xenograft models and treatment with Pen-d/n-ATF5-RP

As described previously [4], for the retrovirus model adult mice were anesthetized and underwent stereotaxic injection of retrovirus expressing PDGF-B and p53-shRNA to generate malignant gliomas. For the xenograft model, 10,000 U87 MG-Luc2 cells (PerkinElmer Health Sciences Inc.), expressing luciferase were stereotactically injected into NOD.Cg-Prkdc^scid^ I/2rg^tm1Wjl^/SzJ (NSG) mice [41]. Analgesics were given immediately after surgery. Injected mice were monitored post-surgically and throughout the study period, which ranged from 13 to 519 days. Pen-d/n-ATF5-RP or Pen-Control-RP was administered to tumor-bearing animals in treatments of four subcutaneous or intraperitoneal injections, spaced 1-2 hours apart. For the retrovirus model, doses were 1 mg/kg (200 μl, 0.9% saline) for each injection. In some experiments as indicated, dosing was repeated 5 days later. Animals injected with 0.9% saline at the same dosing schedule and volume served as controls. For the xenograft model, Pen-d/n-ATF5-RP or Pen-control-RP doses were given one day after the first tumor bioluminescence (> 10^6^ photos per sec flux) in one or two subcutaneous injections, spaced 1 hour apart.

### MRI analysis

Anesthetized (isoflurane and oxygen) mice were fitted intravenously with a 30 gauge catheter, and positioned head first, prone on the scanner bed.

MRI acquisitions were performed on a Bruker Biospec 7 Tesla magnet operating Paravision v5.1 and outfitted with a 116-mm diameter gradient with integrated shim control. Maximum gradient strength was 450mT/m. A cross coil configuration was used for imaging brains and a 72-mm ID linear coil was used for RF transmission and a 4 channel phased array coil for RF reception. Pre-contrast and 1 minute post contrast images were acquired with FLASH_3Dslab. Gadolinium was injected intravenously at a dose of 1μl/g body weight.

FLASH_3Dslab: Fast low angle shot (FLASH) images were acquired with TE 6ms, TR 50-ms and a flip angle of 30 degrees. Spatial resolution was 0.117-mm (read) x 0.117-mm (phase 1) x 0.250-mm (phase 2) with a matrix size of 256×128x64. Motion suppression was enabled. Two averages were taken giving a scan time of 13m, 40s. Amide software determined tumor length and volume by extracting an elliptic cylinder 3-dimensional region of interest from selected voxels. [42].

### Bioluminescence

The NSG mice bearing an U87-MG-luciferase xenograft tumor were anesthetized and then intraperitoneally injected with 150-μl luciferin (30 mg/ml; Perkin-Elmer). The injected mice were placed on a warmed surface in the IVIS (IVIS100 Perkin-Elmer) imaging chamber approximately 3 minutes after injection, and were imaged at 6 and 12 min after injection for qualitative 2-D surface maps representing concentrations of luciferase-expressing tumor cells in the mouse brain.

### Statistical analysis

Student's *t*-test was to measure significant differences between the different treatments. Kaplan-Meier survival analysis achieved by log-rank test Graphpad Prism 6.

### Ethical approval

The methods were carried out in “accordance” with the University of California, Davis approved guidelines under IACUC animal use protocols 16123/17735 and Biological Use Authorization 740. All experimental protocols were approved by University of California, Davis under IUCAC animal use protocols 16123/17735 and Biological Use Authorization 740.

## SUPPLEMENTARY MATERIAL FIGURES AND TABLE


