# Supplementary material for: Regression/Eradication of gliomas in mice by a systemically-deliverable ATF5 dominant-negative peptide
Source: Oncotarget. 2016 Feb 5;7(11):12718–30. doi: 10.18632/oncotarget.7212 (PMC4914317; doi:10.18632/oncotarget.7212)
Supplement: Supplementary file 1 [file oncotarget-07-12718-s001.pdf]

Regression/Eradication of gliomas in mice by a systemically-deliverable ATF5 dominant-negative peptide

Supplementary Material

Methods

Truncation of d/n ATF-5.

Using pQC eGFP-d/n-ATF5 plasmid [1] as template, PCR using upstream primer 5'- TCC GCG GCC GCA CCG GTC GCC -3' and downstream primer 5'- CTC GAG GAT ATC TCA GTT ATC TAC ACT GAC TCT GCC CTC TCC CTC AG -3' truncated 75 base pairs from the 3' plasmid. Electrophoretically purified eGFP-d/n ATF5-tr (tr=truncated) cDNA was ligated into pGEM-T Easy Cloning vector (Promega), transformed into DH5α cells, and plated onto LB agar-Ampicillin plates with blue-white selection. Selected colonies were amplified overnight in LB plus ampicillin. Plasmids isolated from the culture (mini-prep, Invitrogen) were digested with AgeI and EcoRV followed by agarose gel electrophoresis to verify d/n-ATF5-tr insertion and inserts underwent DNA sequencing for verification. AgeI/EcoRV digested eGFP-d/n-ATF5-tr cDNA was ligated into AgeI/EcoRV-digested purified pQCXIX (Clontech) expression vector. The ligation mixture was used to transform DH5α bacteria and the product was verified by AgeI/EcoRV digestion and gel electrophoresis from minipreps of bacterial cultures and DNA sequencing of uncut plasmid. The pQC-*eGFP-d/n-ATF5-tr* plasmid was grown in Maxiprep (Invitrogen).

Bioactivity of the pQC-eGFP-d/n ATF-5tr product (C-terminally truncated d/n-ATF5).

Purified pQC-*eGFP-d/n-ATF5-tr*, full-length pQC-*eGFP-d/n-ATF5* positive control or pQC-*eGFP* negative control plasmids were transfected into rat C6 glial cells in 24 well plates using Lipofectamine 2000 (Invitrogen). After 48 hours, cells were stained with DAPI and 10 random fields were viewed under fluorescent microscopy at 40x. Cells displaying fragmented, condensed chromatin were scored as apoptotic and quantified relative to total cells (n = 3 independent experiments).

Cell penetrating (CP)-6xHis-Pen-Flag-tagged-d/n-ATF5 bioassay

For peptide bioassays, rat C6 glioblastoma cells were maintained in serum-free DMEM for 2 hours, and then in DMEM/0.5% FBS without or with 3 µM Penetratin (Pen)-d/n-ATF5-RP peptide or (Penetratin) Pen-control-RP. After 5 days, cells were stained with DAPI and percent of apoptotic cells determined as described above. For the Pen-d/n-ATF5-SP, flow cytometry was performed as described [2].

Imaging of internalized Pen-d/n-ATF5-RP (Recombinant Protein).

Rat C6 cells (from Jeff Bruce; Columbia University, New York; authenticated 2004 by grafting into Rat brain [1]) and U87 cells (purchased and authenticated from the ATCC) were plated on fibronectin-coated confocal microscopy coverslips and maintained overnight. 3 µM each of Pen-d/n-ATF5-RP or Pen-Control-RP were added to wells and incubated for 1, 2, 4, or 24 hours. Cells were washed 3x with PBS to remove extracellular peptide and stained with primary mouse anti-FLAG antibody (Sigma-Aldrich) overnight followed by incubation for two hours with secondary anti-mouse Alexa-568

(Invitrogen). Microscopy used a Carl Zeiss Axiovert 200 with Axiocam video capture or Delta Vision Deconvolution microscope at 0.1-µm optical sections enhanced by Huygens Deconvolution Software. Images of xy and yz planes confirmed co-localization of Pen-d/n-ATF5-RP and DAPI staining.

Brain sectioning and staining.

As previously described, [3] mice were euthanized by deep isoflurane anesthesia followed by trans-cardial perfusion with 10% formalin. Brains were fixed in 4% paraformaldehyde, incubated overnight in 30% sucrose and were mounted in OCT medium, frozen and cut into 14-µm coronal sections. In other cases as indicated, brains of perfused mice were incubated in 10% formalin/PBS for 4-7 days and then paraffin-embedded. Paraffin sections were subjected to antigen retrieval as described [4]. Sections were stained with DAPI and the following: Anti-Flag M2 (1:200; Sigma-Aldrich), rabbit anti-Flag (1: 1000, Cell Signaling), rabbit anti-HA (4 µg/ml; sc-805 Santa Cruz Biotechnology), or TUNEL (Roche) and Anti-Flag M2. Sections were visualized with a DAPI filter and immunofluorescence (Alexa 488/568; Invitrogen) or colorimetrically with diaminobenzidine or fast red (Mach2; Biocare Medical) and photographed on a Carl Zeiss Axiovert 200 with Axiocam video.

CP-6xHis-Pen-Flag-tagged-d/n-ATF5 protein production and bioassay.

To create *Cell-Penetrating-6xHis-Penetratin-Flag-tagged-d/n-ATF5-tr (CP-6xHis-Pen-Flag-tagged-d/n-ATF5-tr) cDNA*, PCR was first employed using upstream primer 5'- TTA ATT AAG CCG CCA TGG ATG CGT CAA ATT AAA ATT TGG

TTT CAA AAT CGT CGT ATG AAA TGG AAA AAA ATG GAC TAC AAG GAC GAT GAT -3' and downstream primer 5'- CTC GAG GGA TCC TCA GTT ATC TAC ACT GAC TCT GCC CTC TCC CTC AG -3' and pQC-*Flag-d/n-ATF5-tr* as template. The product was purified after gel electrophoresis and ligated into pGEM-T Easy cloning vector. This was transformed into DH5α cells and for white colony selection. Miniprep clones were digested with EcoRV followed by gel electrophoresis and sequencing of uncut plasmid to verify the insert. To insert a 6xHis tag at the N-terminus, *Pen-d/n-ATF5-RP-tr* was cloned into the pET-15b expression vector (Novagen). Both pET-15b and pGEMT-Pen-d/n-ATF5-RP-tr vectors were digested with Nde-1 and BamH1 and the cut Pen-d/n-ATF5-RP-tr was separated from pGEMT by gel electrophoresis. Likewise, cut pET-15b was separated from the insert by gel electrophoresis. Both pET-15b and Pen-d/n-ATF5-RP-tr were excised from the gel and purified and then ligated using T4 DNA ligase. The ligated material was used to transform DH5α cells and colonies selected. Mini-prepped constructs were digested with Xba-1 and EcoRV to verify the presence of vector and Pen-d/n-ATF5-RP insert using gel electrophoresis. The *pET-15b-Pen-d/n-ATF5-RP* was verified by DNA sequencing for correct orientation and sequence.

To generate CP-6xHis-Pen-Flag-tagged-d/n-ATF5 protein, the expression construct was transformed into BL21 DE3 pLysS cells (Novagen). Colonies were selected and amplified in LB. Peptide production was induced with 1 mM IPTG and verified by SDS-PAGE. Once protein induction was verified, extractions

were accomplished with detergent-based BugBuster master mix system (Novagen). Isolation and purification of the Pen-Flag-tagged-d/n-ATF5 peptide was accomplished using its N-terminal 6xHIS-tag and cobalt spin column system (HisPur; Thermo Fisher). Purified peptide was desalted and buffer-exchanged to PBS using Zeba de-salt spin columns (Thermo Fisher) or G-25 Sephadex (GE Health Care). Desalted protein was sterile-filtered using 0.20 µm polyethersulfone membrane syringe filters (Sarstedt). Lastly, the peptide was concentrated to 1-2 mg/ml using Amicon Ultra-4 centrifugal filter devices (3000 MWCutoff).

A control peptide (Pen-Flag-tagged-Control) was created and produced using the same methodology by eliminating the d/n-ATF5 portion of the construct using PCR upstream primer 5’- CCCGGGCATATGCGTCAAATTAAAATTTGGTTT-3’ and downstream primer 5’-
CTCGAGGGATCCTCAGTTATCTAGTCTGGGTCTCTTCC-3’.

Synthetic Pen-d/n-ATF5-SP: This peptide was manufactured at C S Bio, Inc. 20 Kelly Court Menlo Park, CA, 94025; Web: http://www.csbio.com.

Mass Spectroscopy.

<u>Linear MALDI-TOF analysis for nominal molecular mass measurement</u>: Matrix-assisted laser desorption/ionization (MALDI) measurements were acquired on a MALDI-TOF/TOF mass spectrometer (4700 Proteomics Analyzer, AB Sciex) equipped with a 200 Hz ND-YAG laser source (355 nm). Samples were spotted

onto the MALDI plate with an equivolume of MALDI matrix (sinapinic acid in 50% ACN/0.1% FA, Fluka) and air dried. The instrument was operated at an accelerating voltage of 20 kV. Spectra were taken from signal averaging of 4,000 laser shots. Mass Spectra analyses were performed in positive ion linear mode with a mass range of 10,000-60,000 m/z. Data were further analyzed by Data Explorer 4.5 (AB Sciex).

LC-MS analysis: Samples were injected onto an Aeris Widepore XB-C8 column (3.6 µ, 2.10×50 mm). A standard reverse phase gradient was run over 8 minutes at flow rate of 250 µl/min and the eluent monitored by a LTQ-OrbitrapXL mass spectrometer (Thermo Fisher) in profile mode. Ion Max Source (Thermo Fisher) was used as the electrospray ionization source and source parameters were 5kV spray voltage, capillary temperature of 275°C and sheath gas setting of 20. Spectral data were acquired at a resolution setting of 15,000 FWHM with the lockmass feature.

Apoptosis of Glioma Stem Cells: The treated glioma stem cells were stained with Annexin V (FITC)/Propidium Iodide and the resulting apoptosis was measured by flow cytometry [2].

1. Angelastro JM, Canoll PD, Kuo J, Weicker M, Costa A, Bruce JN and Greene LA. Selective destruction of glioblastoma cells by interference with the activity or expression of ATF5. Oncogene. 2006; 25(6):907-916.
2. Karpel-Massler G, Shu C, Chau L, Banu M, Halatsch ME, Westhoff MA, Ramirez Y, Ross AH, Bruce JN, Canoll P and Siegelin MD. Combined inhibition of Bcl-2/Bcl-xL and Usp9X/Bag3 overcomes apoptotic resistance in glioblastoma in vitro and in vivo. Oncotarget. 2015; 6(16):14507-14521.
3. Arias A, Lame MW, Santarelli L, Hen R, Greene LA and Angelastro JM. Regulated ATF5 loss-of-function in adult mice blocks formation and causes regression/eradication of gliomas. Oncogene. 2012; 31(6):739-751.
4. Schrot RJ, Ma JH, Greco CM, Arias AD and Angelastro JM. Organotypic distribution of stem cell markers in formalin-fixed brain harboring glioblastoma multiforme. J Neurooncol. 2007; 85(2):149-157.

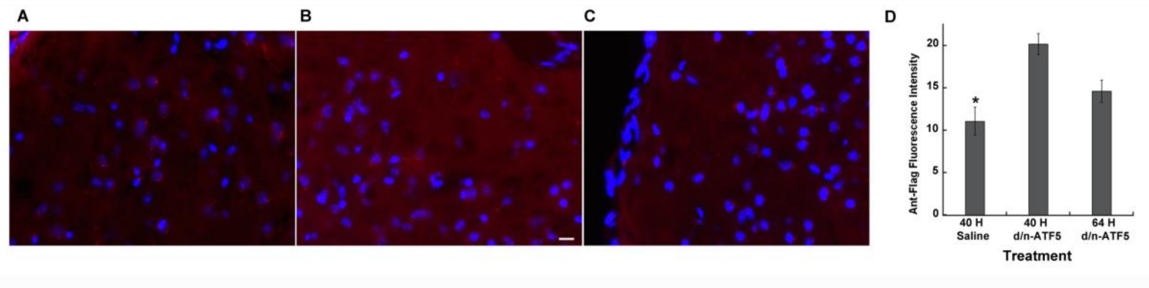

Supplementary Figure S1: Retention of Pen-d/n-ATF5-RP in mouse brain at various times after administration. Mice received 4 intraperitoneal injections of either saline (A) or Pen-d/n-ATF5-RP (B,C) as described in the text. Animals were sacrificed at either 40 (A,B) or 64 (C) h after the last injection and sections of their fixed brains were stained with either anti-Flag (Red; to visualize Pen-d/n-ATF5-RP) or DAPI (blue; to visualize nuclei). (D) Densitometry of anti-Flag Immunostaining. The optical densities (red channel) of fifteen random 0.176 inch^2 areas were determined in each of the images and averaged ± SD using Image J. Student's t-test; Pen-d/n-ATF5-RP (64 hours) or (40 hours) versus saline, (*$p<0.05$). Scale bar is 10 µm.

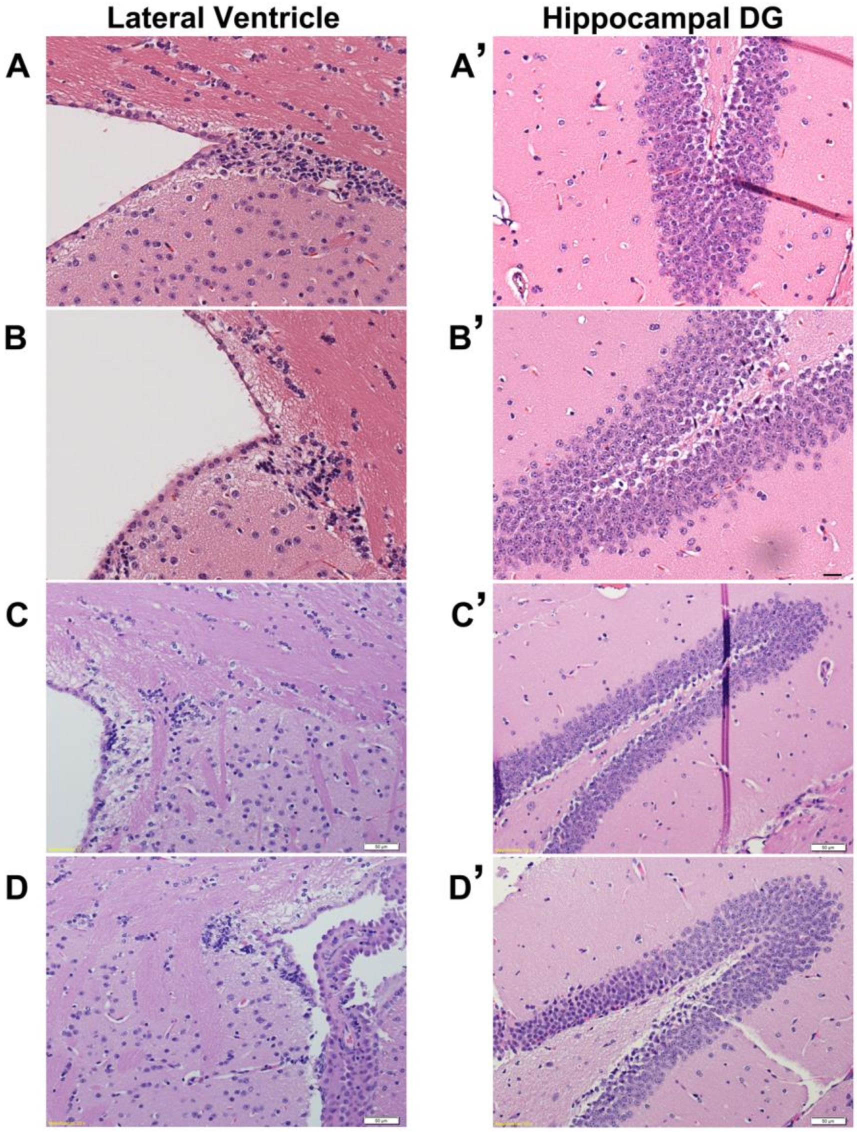

Supplementary Figure S2: H&E staining of the SVZ and hippocampal dentate gyrus shows no detectable difference between these structures in Pen-d/n-ATF5-RP-treated and non-treated mice. (A, A’) Lateral ventricle/SVZ (A) and hippocampal dentate gyrus (A’) from a tumor-bearing mouse 183 days after the second set of subcutaneous treatments with Pen-d/n-ATF5-RP (see also Supplementary Figure 4 for further data on the same mouse). (B, B’) Lateral ventricle/SVZ (B) and hippocampal dentate gyrus (B’) from an age-matched

control mouse not treated with Pen-d/n-ATF5-RP and not injected with retrovirus. (C, C’) Lateral ventricle/SVZ (C) and hippocampal dentate gyrus (C’) from a non-tumor-bearing mouse 1 day after the second set (given 5 days after the first set) of subcutaneous treatments with Pen-d/n-ATF5-RP. (D,D’) Lateral ventricle/SVZ (D) and hippocampal dentate gyrus (D’) from an age-matched untreated non-tumor-bearing control mouse. Scale for A-C is 20 µm and 50 µm for D-F.

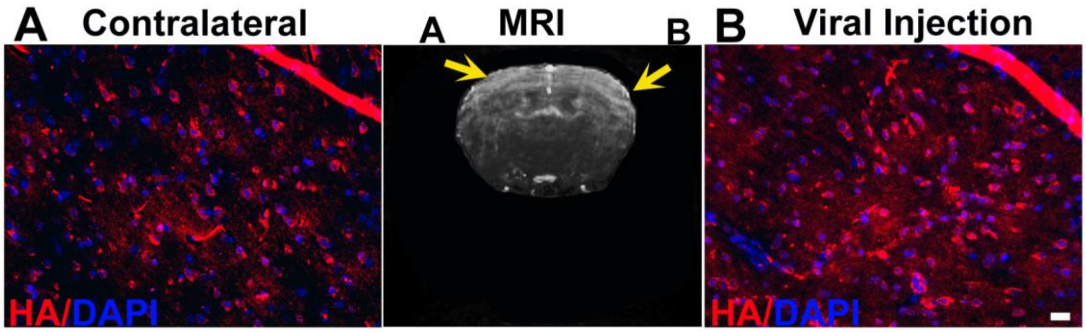

Supplementary Figure S3: MRI and histopathological images of an untreated mouse with a bilateral tumor. Middle panel shows post-contrast 3D FLASH MRI image of a tumor-bearing mouse brain at 112 days after PDGF-B-HA/shp53 retrovirus injection. Panels (A) and (B) show images for sections stained with HA to reveal tumor cells and with DAPI to show nuclei. The yellow arrows on the MRI along with the letters show the relative locations of the HA+ sections shown in (A) and (B). Retroviral injection was on side B. Scale bar is 20 µm. DAPI (4,6-diamidino-2-phenylindole).

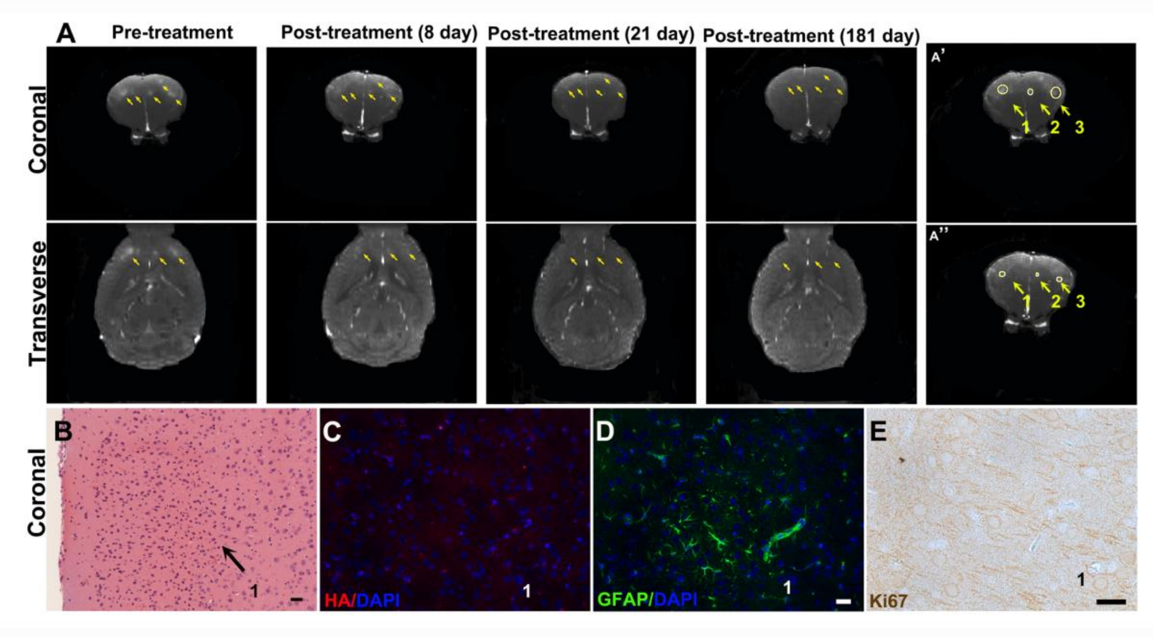

Supplementary Figure S4: Second example illustrating that Pen-d/n-ATF5-RP promotes rapid and long-term regression/eradication of a mouse glioma as indicated by MRI and histology. (A) Post-contrast 3D FLASH MRI images of a tumor-bearing mouse brain before and at various times after treatment with Pen-d/n-ATF5-RP. Pretreatment coronal and transverse images (74 days after PDGF-B-HA/shp53 retrovirus injection) show multifocal tumors within the cortex (arrows). Images from the same mouse brain are shown at 8, 21 and 181 days after two sets of subcutaneous treatments with Pen-d/n-ATF5-RP as described in the text. Note decreased signal at 8 days and absence of detectable signals at 21 and 181 days following treatment. (A', A'') Estimates of tumor volume corroborate loss of signal by 8 days after Pen-d/n-ATF5-RP treatment. The same images as in (A) for pretreatment and 8 days post-treatment with arrows pointing to tumor foci (yellow circles) for which volumetric measurements were obtained using the region of interest elliptic cylinder tool (yellow circles). At

pretreatment, the calculated volumes in (A’) are 0.597 mm^3, 0.164 mm^3, and 0.760 mm^3 for foci 1, 2, and 3, respectively. For 8 days post-treatment (A’’), volumes of the same tumors decreased to 0.106 mm^3, 0.0302 mm^3, and 0.0895 mm^3 for foci 1, 2, and 3, respectively. After 21 days of treatment the tumors could not be visualized for measurement. (B) H&E staining of the same sacrificed mouse brain (183 days after treatment; 190 days after initial tumor detection) corroborates the absence of detectable tumor with the arrow pointing to the remnant scar corresponding to tumor focus 1 shown in A’ and corroborates absence of detectable tumor. (C) HA immunostaining of the same brain (for PDGF-B-HA) indicates the absence of detectable tumor cells in the same focus 1 region as in (A’) and (B). (D) GFAP immunostaining of the same brain at focus 1 shows a remnant GFAP+ glial scar. (E) Ki67 immunostaining of the focus 1 region of the same brain reveals the absence of dividing cells. Scale bar is 20 µm for B-E.

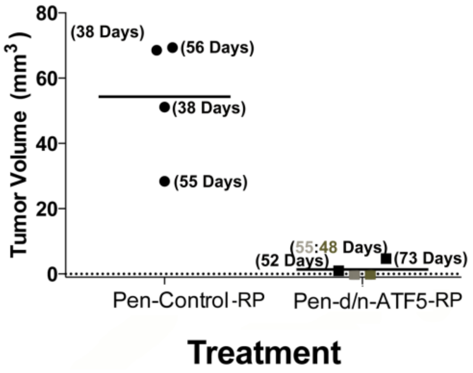

Supplementary Figure S5: Second Tumor Model with U87-MG-Luc2 (expressing luciferase) intracerebral xenografts in NSG mice. Non-invasive bioluminescence imaging monitored tumor growth and formation for each mouse and post-histological examination of the brain and the tumors was achieved after the moribund behavior endpoint or 20% body weight loss. Treatment time-course began one day after initial detection of the tumor at $\geq 10^6$ photos/sec. Various treatment regimens at 4 mg/kg ranged from two delivery's at 5 days apart, then one week, and then two weeks later (n=3 for both Pen-Control and Pen-d/n-ATF5). Except two mice received treatment 5 days apart, then one week, then 3 weeks later (Pen-Control and Pen-d/n-ATF5), with one mouse of the two having a final dose 5 weeks later (Pen-d/n-ATF5). Unpaired t-test assuming equal standard deviations determined significant difference (p=0.0016) for Pen-Control (n=4) and Pen-d/n-ATF5 (n=4). Mice were sacrificed when moribund behavior was shown. Tumor volume was determined post-histological with H&E stained slides. The volume was determined by elliptic cylinder tool in ImageJ. Area was first determined and volume was measured by the thickness of the tissue slice. Data are tumor volume against dosage and the day when moribund behavior was detected (Day since detection).

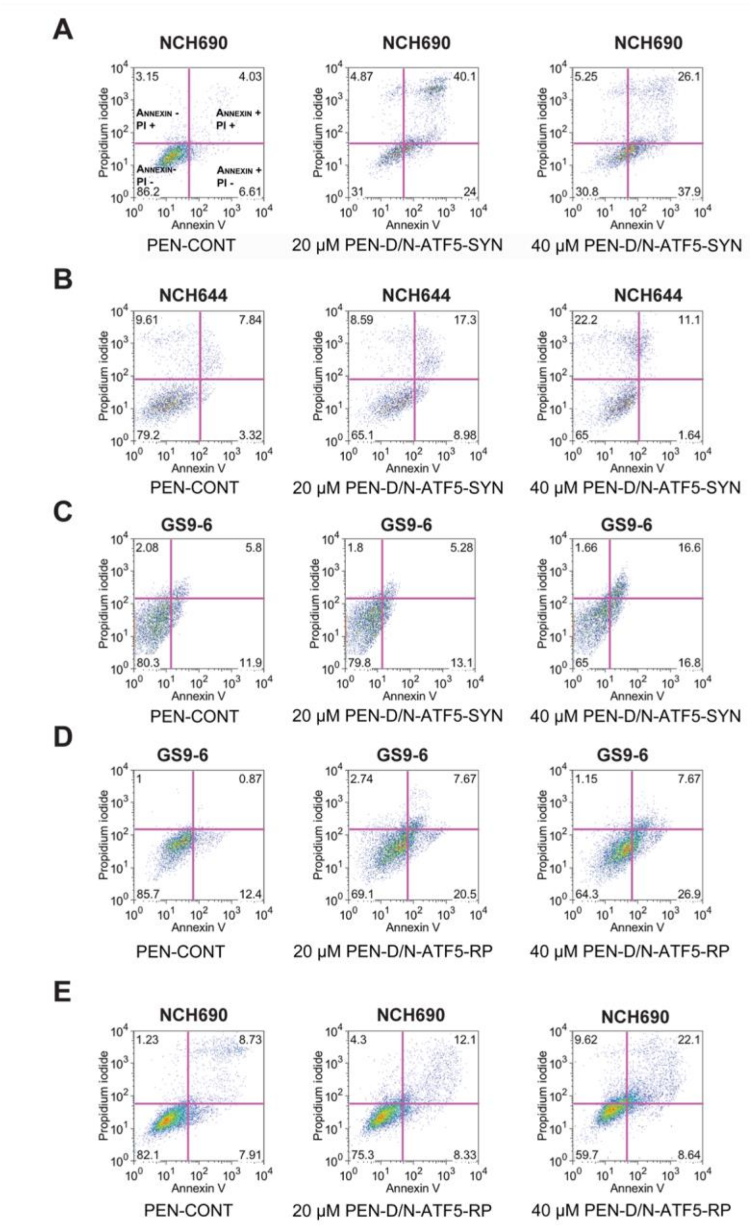

Supplementary Figure S6: CP-d/n-ATF5 elicits apoptotic cell death in stem cell-like glioblastoma cell cultures (GICs: Glioma initiating cells). (A-C) Stem-cell like glioblastoma cell cultures, NCH690 (A), NCH644 (B) and GS9-6 (C) were treated with 20 µM recombinant Penetratin control or with synthetic Penetratin-d/n-ATF5-SYN (20 and 40 µM). After 5 days of treatment cells were stained with Annexin V (FITC) and Propidium iodide and analyzed by multi-parametric flow cytometry.

(D-E) Stem-cell like glioblastoma cultures, GS9-6 and NCH690 were treated with 20 µM recombinant Penetratin control or with recombinant Penetratin-linked CP-d/n-ATF5-RP (20 and 40 µM). After 5 days of treatment cells were stained with Annexin V (FITC) and Propidium iodide and analyzed by multi-parametric flow cytometry.

Table SI: Results from gross necropsy of organs, H&E staining of tissue sections and liver-kidney function blood panel of mice treated with Pen-d/n-ATF5-RP.

**Gross Necropsy/ H&E slides**	**1 day after Pen-d/n-ATF5-RP treatment #1**	**1 day after Pen-d/n-ATF5-RP treatment #2**	**Control**	**2 day after Pen-d/n-ATF5-RP treatment #1***	**2 day after Pen-d/n-ATF5-RP treatment #2***	**6 Month Post-tumor treatment with Pen-d/n-ATF5-RP #1**	**6 Month Post-tumor treatment with Pen-d/n-ATF5-RP #2**
Cerebrum, cerebellum, nasal cavity, liver, kidneys, spleen, pancreas, heart, lungs, trachea, esophagus, thymus, salivary glands, GI tract, hind limb muscles, urinary bladder, reproductive tract	No gross lesion/No significant changes of pathological significance	No gross lesion/No significant changes of pathological significance	No gross lesion/No significant changes of pathological significance	No gross lesion/No significant changes of pathological significance	No gross lesion/No significant changes of pathological significance	No gross lesion/No significant changes of pathological significance	No gross lesion/No significant changes of pathological significance

Liver-kidney function panel.

Mouse	Albumin g/dL	Alkaline Phosphatase U/L	Alanine Transaminase U/L	Asparate Transaminase U/L	Blood Urea Nitrogen mg/dL	Creatinine mg/dL	Total Bilirubin mg/dL	Total Protein g/dL	Lipemia	Hemolysis
Male, one day after Pen-d/n-ATF5-RP treatment #1	3.95	52.9	55.4	128.7	20.4	0.072	0.089	5.87	None	None
Male, one day after Pen-d/n-ATF5-RP treatment #2	3.35	46.0	21.6	66.6	19.7	0.061	0.115	5.21	None	None
JAX database strain range (males)	3.77± 0.247	78.3± 32.6	52.7± 19.6	152± 92.6	23.7 ± 3.47	0.167± 0.258	0.695± 0.167	6.10± 0.396	Not Listed	Not Listed

Legend: Necropsy, H&E tissue staining and liver-kidney function panel reveal no short- or long-term pathological responses to treatment with Pen-d/n-ATF5-RP.

The indicated organs were collected from mice sacrificed at 1 day, 2 days and > 6months (192 days and 183 days, corresponding to mice with eradicated tumors in Figure 7, and Supp Figure 4, respectively) after the second of two sets of subcutaneous treatments with Pen-d/n-ATF5-RP as described in the text. The >6 month animals had MRI-detected tumors before treatment and no histologically detectable tumors at the time of sacrifice. All other animals were not tumor-bearing. The control mouse was untreated. The organs were evaluated for gross pathological changes and then fixed, paraffin embedded and used for preparation of slide-mounted 5 µm sections. The slides were stained with H&E and examined microscopically for possible pathological changes. Gross pathological analysis and evaluation of sections were carried out by the Comparative Pathology Laboratory at the UC Davis School of Veterinary Medicine. *Regional coagulative necrosis in the liver and focal linear pneumonia of the lung were observed due to inadvertent needle penetration during the injections.

For liver-kidney function panel, blood samples were obtained 1 day after the second of two sets of subcutaneous treatments with Pen-d/n-ATF5-RP as described in the text. The animals were not tumor-bearing. The analysis was carried out by the Comparative Pathology Laboratory at the UC Davis School of Veterinary Medicine. The data for the strain (C57BL/6J) range was obtained from the Mouse Phenome Database at the Jackson Laboratory (http://phenome.jax.org/db/q?rtn=meas/catlister&req=Dblood--clinical%20chemistryqqq44&reqstrainid=7).
